# Phyto-Mediated Synthesis of Porous Titanium Dioxide Nanoparticles From *Withania somnifera* Root Extract: Broad-Spectrum Attenuation of Biofilm and Cytotoxic Properties Against HepG2 Cell Lines

**DOI:** 10.3389/fmicb.2020.01680

**Published:** 2020-07-28

**Authors:** Nasser A. Al-Shabib, Fohad Mabood Husain, Faizan Abul Qais, Naushad Ahmad, Altaf Khan, Abdullah A. Alyousef, Mohammed Arshad, Saba Noor, Javed Masood Khan, Pravej Alam, Thamer H. Albalawi, Syed Ali Shahzad

**Affiliations:** ^1^Department of Food Science and Nutrition, College of Food and Agricultural Sciences, King Saud University, Riyadh, Saudi Arabia; ^2^Department of Agricultural Microbiology, Aligarh Muslim University, Aligarh, India; ^3^Department of Chemistry, College of Sciences, King Saud University, Riyadh, Saudi Arabia; ^4^Department of Pharmacology and Toxicology, Central Laboratory, College of Pharmacy, King Saud University, Riyadh, Saudi Arabia; ^5^Department of Clinical Laboratory Sciences, College of Applied Medical Sciences, King Saud University, Riyadh, Saudi Arabia; ^6^National Institute of Cancer Prevention and Research, Noida, India; ^7^Department of Biology, College of Science and Humanities, Prince Sattam Bin Abdulaziz University, Al-Kharj, Saudi Arabia

**Keywords:** TiO_2_ NPs, green synthesis, *Withania somnifera*, antibiofilm, HepG2, cytotoxicity

## Abstract

There is grave necessity to counter the menace of drug-resistant biofilms of pathogens using nanomaterials. Moreover, we need to produce nanoparticles (NPs) using inexpensive clean biological approaches that demonstrate broad-spectrum inhibition of microbial biofilms and cytotoxicity against HepG2 cell lines. In the current research work, titanium dioxide (TiO_2_) NPs were fabricated through an environmentally friendly green process using the root extract of *Withania somnifera* as the stabilizing and reducing agent to examine its antibiofilm and anticancer potential. Further, X-ray diffraction (XRD), Fourier transform infrared (FTIR), scanning electron microscopy (SEM), transmission electron micrograph (TEM), energy-dispersive X-ray spectroscopy (EDS), dynamic light scattering (DLS), thermogravimetric analysis (TGA), and Brunauer-Emmett-Teller (BET) techniques were used for determining the crystallinity, functional groups involved, shape, size, thermal behavior, surface area, and porosity measurement, respectively, of the synthesized TiO_2_ NPs. Antimicrobial potential of the TiO_2_ NPs was determined by evaluating the minimum inhibitory concentration (MIC) against *Escherichia coli*, *Pseudomonas aeruginosa*, methicillin-resistant *Staphylococcus aureus*, *Listeria monocytogenes*, *Serratia marcescens*, and *Candida albicans*. Furthermore, at levels below the MIC (0.5 × MIC), TiO_2_ NPs demonstrated significant inhibition of biofilm formation (43–71%) and mature biofilms (24–64%) in all test pathogens. Cell death due to enhanced reactive oxygen species (ROS) production could be responsible for the impaired biofilm production in TiO_2_ NP–treated pathogens. The synthesized NPs induced considerable reduction in the viability of HepG2 *in vitro* and could prove effective in controlling liver cancer. In summary, the green synthesized TiO_2_ NPs demonstrate multifarious biological properties and could be used as an anti-infective agent to treat biofilm-based infections and cancer.

## Introduction

In the last decade, the world has witnessed tremendous advancements in the field of nanoscience and its applicability in diverse domains, including academics, industry, and medicine. The distinct physicochemical characteristics and high surface area-to-volume ratio of nanoparticles (NPs) make them attractive candidates for the development of biocompatible materials that can be used in industries and clinical settings (Eisa et al., 2019). Metallic NPs with desired properties have been synthesized using several physical and chemical methods; however, these methods are expensive, utilize hazardous chemicals, require high levels of energy, and expel toxic byproducts that are deleterious to the environment (Patra and Baek, 2015). Therefore, we need methods that exert minimum risk on the environment and also are economically cost effective.

In recent years, bioinspired fabrication of NPs using various biological systems, such as microorganisms (Oves et al., 2019) and plants (Al-Shabib et al., 2016, 2018b), has gained momentum. The plant-mediated NP synthesis has generated lot of interest due to the wide availability of the plants; safe, clean, and eco-friendly synthesis; and low energy consumption (Rajkumari et al., 2019). Aqueous extracts of various plant parts, including seeds, roots, leaves, stems, and fruits have been used for metallic NP synthesis. The phytoconstituents in the extract act as reducing and stabilizing agents for non-toxic NP production (Siddiqi et al., 2018).

The phytomediated synthesis of titanium oxide (TiO_2_) NPs has great potential in producing anti-infective agents. Titanium oxide NPs are documented to be safe, stable, non-toxic, and having surface activity; hence, they are among the most widely used nanomaterials. The biomediated production of TiO_2_ NPs has found application in disease treatment, surgical product manufacture, photocatalysis, tissue engineering, agriculture, and cosmetics (Nadeem et al., 2018). Various plants and their parts have been reported for the production of TiO_2_ NPs, including *Acanthophyllum laxiusculum* (roots), *Aloe barbadensis* (leaves), *Annona squamosa* (peel), *Calotropis gigantea* (flower), *Cicer arietinum* (seeds), and *Dandelion* (pollen) (Marimuthu et al., 2013; Bao et al., 2012; Roopan et al., 2012; Kashale et al., 2016; Madadi and Lotfabad, 2016; Rajkumari et al., 2019).

*Withania somnifera*, a member of the solanaceae family, is a well-known medicinal plant in Ayurvedic and Unani system of medicine, commonly called as Ashwagandha. The plant has been documented to exhibit medicinal benefits against several ailments, including neurodegenerative diseases, cancer, and chronic diseases The antibacterial activity of *W. somnifera* has been explored for many decades. Studies have shown that its extracts demonstrated bactericidal potential against methicillin-resistant *Staphylococcus aureus*, *Streptococcus pyogenes*, *Enterococcus faecalis*, *Klebsiella pneumoniae*, and *Escherichia coli* (Rizwana, 2012). To date, numerous researchers have synthesized NPs using *W. somnifera*. For instance, silver NPs synthesized from the aqueous extract of *W. somnifera* exhibited broad-spectrum antibacterial and antibiofilm activity. A study reported its multiple modes of action, including microbial growth inhibition, cell membrane damage, and reactive oxygen species (ROS) production (Qais et al., 2018). Studies on TiO_2_ NP synthesis from *W. somnifera* are still scarce, and this probably is the first report on TiO_2_ NP synthesis from this plant.

In most natural environments, bacteria and fungi prefer to grow in biofilm mode. Microbial biofilms are a complex ecosystem comprising of one or more species embedded in an exopolysaccharide (EPS) matrix (Galié et al., 2018). Formation of biofilm starts with the adherence of the cells to an inert surface and culminates by the formation of cell clusters embedded in EPS matrix secreted by the microbe (Johnson, 2008). Biofilm control and eradication are a major area of concern for environmentalists, food technologists, and clinicians, as it manifests the microbial community resistant to antimicrobials and disinfectants (Baptista et al., 2018). Further, drug-resistant biofilms on medical implants, such as catheters, sutures, and dental implants, lead to severe persistent infection. Further, it makes the treatment more expensive and harassing for the patient (Costerton et al., 2005). Biofilm structures are formed on different artificial surfaces in the food industry, such as stainless steel, glass, and rubber. This leads to pathogenicity, corrosion of metal surfaces, and organoleptic property alteration, which is critical to various agro-based industries (Galié et al., 2018). In addition to conferring resistance to microbes, reports indicated that biofilm formation has a potential etiologic role in cancer development (Rizzato et al., 2019). Experimental evidence has suggested that cancer initiation and development may be a consequence of the pro-oncogenic properties of biofilms formed by invasive pathogenic bacteria (Johnson et al., 2015).

Considering the deleterious effects of biofilms in infections and cancer, we synthesized TiO_2_ NPs from the root extract of *W. somnifera* and characterized them using various spectroscopic and microscopic techniques. Further, we studied its broad-spectrum antibiofilm potential against *E. coli*, *Pseudomonas aeruginosa*, methicillin-resistant *S. aureus*, *Listeria monocytogenes*, *Serratia marcescens*, and *Candida albicans*. In addition, we also explored the effects of newly synthesized TiO_2_ NPs on human liver cancer cell line HepG2.

## Materials and Methods

### Collection of Plant Sample and Preparation of Aqueous Extract

The root of *W. somnifera* was obtained from The Himalaya Drug Company, Dehradun, India. The authentication and identification of the plant material were done at Himalaya Drug Company, as well as at the Department of Botany, AMU, Aligarh, and a voucher specimen (WS/R-AGM/HDCO/01-2017) is submitted at the Department of Agricultural Microbiology, AMU, Aligarh, India. A 5% aqueous extract was prepared in of double-distilled water by heating at 100°C for 1 h. The suspension was centrifuged (15,000 × *g* for 10 min) and filtered to obtain the extract.

### Synthesis of Porous TiO_2_ Nanoparticles (TiO_2_ NPs)

The fine root powder of *W. somnifera* was washed and dried and then used to make aqueous extract by boiling. The synthesis of TiO_2_ NPs was carried out using aqueous extract of *W. somnifera* by previously method with slight modifications (Velayutham et al., 2012). The obtained root extract was mixed with titanium (IV) oxide (5 mM) in a round-bottom flask under constant stirring. In the reaction mixture, 1 mM NaOH was added drop-wise and stirred at 70°C for 3 h. The as-prepared white TiO_2_ NPs NPs were separated by centrifugation (15,000 × *g*, 20 min), washed thrice with distilled water and then with ethanol, and dried overnight at 120°C to obtain porous fine powder that was further characterized by various structural and morphological techniques. The synthesis of nanomaterial via green route is simple, efficient, facile, inexpensive, and ecofriendly that does not require any special condition, such as vacuum, urbane instrument, catalyst, or template, and so on.

### Characterization

X-ray diffraction (XRD) measurements were conducted using a Rigaku Ultima IV diffractometer (Japan) with CuKα radiation (α = 1.54056 Å). Fourier transform infrared (FTIR) spectra were measured with a JASCO spectrometer 4100 (United States) using the KBr pellet technique. The thermal stability of the porous sample was studied using Mettler Toledo thermogravimetric analysis (TGA)/DSC 1 STARe thermogravimetric analyzer (Switzerland) between 50°C and 900°C. The porosity and Brunauer-Emmett-Teller (BET) surface area measurement of the sample were measured at liquid nitrogen temperature with a Micromeritics TriStar 3000 analyzer (Germany) at 77 K. Pretreatment of the samples was done at 200°C for 3 h under high vacuum. Pore-size distributions were calculated using the BJH model on the adsorption branch. Transmission electron micrographs (TEMs) were obtained using a JEOL 2010 microscope (United States) operating at an accelerating voltage of 80 kV. The sample was prepared by placing and evaporating a drop of the sample in ethanol on a carbon-coated gold grid. Scanning electron microscopy (SEM)–energy-dispersive X-ray spectroscopy (EDS) of TiO_2_ NPs was examined by scanning electron microscopy (SEM, JSM-7001F; JEOL, United States) equipped with EDS. The NP size distribution and zeta potential results were carried out by dynamic light scattering (DLS) on Malvern zeta potential/particle size analyzer (United Kingdom). The zeta potential values were obtained by applying the Helmholtz–Smoluchowski equation built into the Malvern. Prior to the measurement, 10 mg of the sample was sonicated in distilled water for 10 min. The measurements were repeated three times for each sample.

### Microbial Strains and Growth Conditions

Six pathogens, namely, *E. coli* ATCC 35218, *P. aeruginosa* ATCC 27853, methicillin-resistant *S. aureus* (MRSA) ATCC 43300, *L. monocytogenes* ATCC 19114, *S. marcescens* ATCC 13880, and *C. albicans* ATCC 10231 were used. All bacteria were preserved on nutrient agar at 4°C, and the lone fungus used in the study was maintained in Sabouraud dextrose agar. Bacterial strains were cultured on nutrient broth at 37°C, whereas Sabouraud dextrose broth was used to grow *C. albicans*.

### Determination of Minimum Inhibitory Concentration and Minimum Bactericidal Concentration

The broth microdilution method using TTC (2,3,5-triphenyl tetrazolium chloride) was adopted to determine the minimum inhibitory concentration (MIC) of TiO_2_ NPs against all test pathogens. Further, minimum bactericidal concentration (MBC) was determined by macrobroth dilution assay. Test pathogens were grown overnight in media containing TiO_2_ NPs concentrations (0.5–256 μg/mL) (Qais et al., 2019).

### Biofilm Inhibition Assay

Overnight-grown pathogens were diluted in wells containing fresh tryptic soy broth and respective 0.5 × MIC (*E. coli*: 16, *P. aeruginosa*: 32, *L. monocytogenes*: 64, *S. marcescens*: 8, methicillin-resistant *S. aureus*: 32, and *C. albicans*: 64 μg/mL) of TiO_2_ NPs and incubated at 37°C for a day. After 24 h, the broth was decanted, and the wells were rinsed three times. The cells attached in each well were stained with 1% crystal violet. After 15 min, stain was decanted, and the wells were washed to remove excess stain. Stain was dissolved in ethanol (200 μL), and absorbance was read at 585 nm to determine inhibition (Hasan et al., 2019).

### Microscopic Analysis of Biofilm Inhibition

Biofilm inhibition upon treatment with 0.5 × MIC of TiO_2_ NPs was observed under confocal laser scanning microscope (CLSM) as previously described (Rajkumari et al., 2019).

### Quantification of EPSs

Test bacteria cultured in the presence and absence of 0.5 × MIC of TiO_2_ NPs were centrifuged, and the filtered supernatant was mixed with chilled ethanol and incubated at 4°C for 18 h to precipitate the EPSs. Phenol–sulfuric acid method to estimate sugars was employed to quantify EPS (Al-Shabib et al., 2018a).

### Protein Leakage Assay

In 10 mL growth media, test bacteria, and NPs were added in such a way that the final concentration attained was 0.5 × MIC. The cells were incubated at 37°C with 150 revolutions/min shaking and examined at 0 and 4 h to determine protein leakage. Incubated cells were centrifuged at 18,000 × *g*, and the resulting supernatants were stored at −20°C. Bradford reagent was used to determine the protein concentration in the supernatants (Qayyum et al., 2017).

## Disruption of Mature Biofilms

Biofilms of the test pathogens were allowed to grow for 24 h in the wells of a microtiter plate. After incubation, media containing unattached cells was discarded, and adhering cells were incubated again for 24 h in media amended with 0.5 × MIC of TiO_2_ NPs. Non-adhering cells were washed with sterile water, and cells bound to walls of the well were stained with crystal violet for 15 min. Absorbance was read at 585 nm after removing the excess stain (Al-Shabib et al., 2018a).

### Effect on ROS Production

Intracellularly produced ROS in the test pathogens that were untreated or treated with TiO_2_ NPs was determined using an oxidation-sensitive fluorescent probe, 2,7-dichlorofluoroscein diacetate (Qais et al., 2018). The experiment was performed at the respective 0.5 × MICs (*E. coli*: 16, *P. aeruginosa*: 32, *L. monocytogenes*: 64, *S. marcescens*: 8, methicillin-resistant *S. aureus*: 32, and *C. albicans*: 64 μg/mL).

### Cytotoxicity Assessment of TiO_2_ NPs

HepG2 (ATCC HB-8065) human hepatocellular carcinoma cells and HEK-293 (ATCC CRL-1573) human embryonic kidney cells were obtained from the American Type Culture Collection (Manassas, VA, United States), cultured in Dulbecco modified eagle medium supplemented with 10% fetal bovine serum, 0.2% sodium bicarbonate, and antibiotics at 37°C under humid condition with 5% carbon dioxide, and were used to assess the anticancer potential of TiO_2_ NPs. HepG2 cells with 98% viability and passage numbers 20 and 22 were selected for dimethylthiazol diphenyltetrazolium bromide (MTT) and the neutral red uptake (NRU) assays.

### MTT Assay

The cell viability of TiO_2_ NP–treated HepG2 and non-tumorigenic HEK-293 cells was assessed using yellow dye MTT. Furthermore, cells (10^4^) were seeded in 96-well microtiter plate and kept in a CO_2_ incubator for 24 h for adherence. Different concentrations of TiO_2_ NPs were added, and plate was incubated for another 24 h. MTT (10 μL) was added to each well, and the reaction mixture was kept for 4 h. Dimethyl sulfoxide (200 μL) was added after discarding the supernatant, and absorbance was read at 550 nm (Farshori et al., 2014).

### NRU Assay

Neutral red uptake assay was also executed to assess the cytotoxicity employing an earlier reported protocol (Al-Ajmi et al., 2018). Concisely, the medium was aspirated with TiO_2_ NPs posttreatment; the HepG2 cells were washed twice and left for 3-h incubation in a medium containing neutral red (50 μg/mL). Solution comprising 0.5% formaldehyde and 1% calcium chloride was used to wash the reaction mixture. The dye was extracted by incubating the cells in a mixture of ethanol (50%) and acetic acid (1%) for 20 min at 37°C. Absorbance was measured at 540 nm.

### Statistical Analysis

All experiments were done in triplicate, and data are presented as mean values. The level of significance was analyzed using Student *t* test in Sigma Plot 12.

## Results and Discussion

### Synthesis and Characterization of TiO_2_ NPs

**FIGURE 1:**
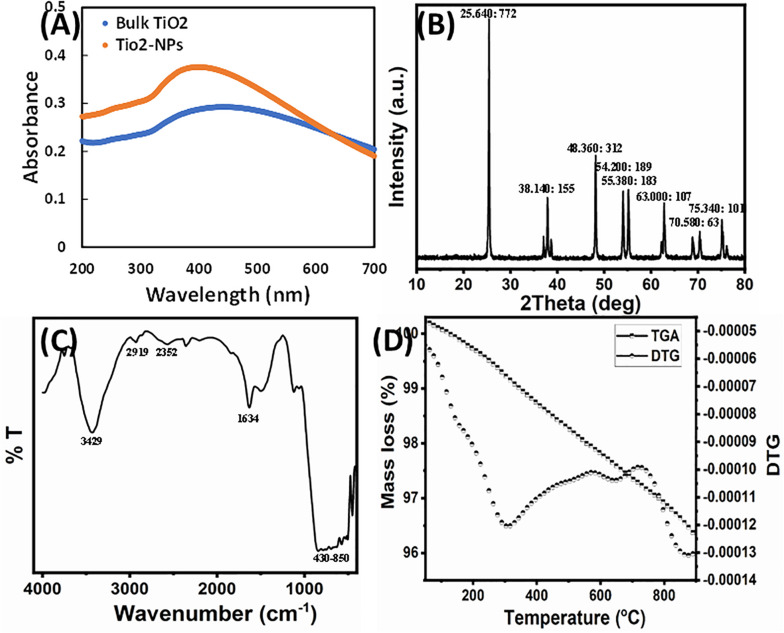
**(A)** UV-Vis spectra of bulk TiO_2_ and TiO_2_ NPs. **(B)** X-ray powder diffraction patterns of the TiO_2_ NPs. Peak information is provided as (2θ: intensity). **(C)** FTIR spectrum of TiO_2_ NPs. **(D)** TG-DTG decomposition curve of TiO_2_ NPs.

The aqueous extract of *W. somnifera* root contains several major active phytochemicals such as withanolides, sitoindosides, amino acids, alkaloids, phenolic compounds, flavonoids, and several other bioactive metabolites (Dar et al., 2015). These compounds act as the stabilizing/capping agent for TiO_2_ NPs biosynthesis. Synthesized NPs were characterized using XRD, FTIR, DLS, TGA, BET surface area, SEM, and TEM techniques. In this study, a green approach was deployed for the TiO_2_ NPs synthesis using *W. somnifera* extract at room temperature. The TiO_2_ suspension was whitish, which changed to light green by the addition of extract, indicating the formation of TiO_2_ NPs. The primary characterization of TiO_2_ NPs synthesis was done recording the UV-Vis spectra. The bulk TiO_2_ showed an absorbance maximum at 440 nm, which blue-shifted to 395 nm after 6 h of reaction, advising the formation of NPs as shown in Figure 1A (Kirthi et al., 2011). This agrees with a previous finding where TiO_2_ NPs synthesis was confirmed by an absorption peak at 380 nm of UV-Vis spectrum (Murugan et al., 2016).

The phase structure of the synthesized TiO_2_ NPs was characterized by XRD. Figure 1B presents the XRD pattern of the synthesized sample. The diffraction peaks in the prepared sample can be indexed to the anatase structure phase (JCPDS card no. 21-1272). The synthesized TiO_2_ NPs exhibited diffraction peaks at 25.64°, 37.07°, 37.90°, 38.73°, 48.21°, 53.89°, 55.19°, 62.72°, 68.93°, 70.47°, 75.22°, and 76.28°. No feature peaks of rutile (27.45°) were observed, and the Brookite form of TiO_2_ NPs and extract/other compounds appeared in the sample. The average crystallite size of the NPs sample was calculated from the anatase FWHM (25.64°) reflection plane using Scherrer formula and found to be 45.28 nm. The anatase diffraction planes were sharp, indicating good TiO_2_ NP crystallization.

Fourier transform infrared analysis was performed to assess the role of various phytoconstituents, mainly functional groups, of presence in the extract that were responsible for the capping and stabilization of TiO_2_ NPs. The FTIR spectrum of synthesized TiO_2_ NPs is shown in Figure 1C. A broad and consistent band in IR spectrum of TiO_2_ NPs from 430 to 850 cm^–1^ corresponds to the vibration of metal–oxygen (Yan et al., 2004; Amlouk et al., 2006). Moreover, prominent peaks in the 450- to 800-cm^–1^ range are due to Ti–O and Ti–O–O stretching vibrations, confirming the formation of TiO_2_ NPs (Sankar et al., 2014; Rajakumar et al., 2015). The formation of TiO_2_ NPs was also confirmed by the absorption band near 547 cm^–1^ that corresponds to Ti–O bond (Zhang et al., 2003; Kang et al., 2009). A broadband nearly at 3,429 cm^–1^ is because of the O–H stretching vibration of the interlayer physically absorbing water molecules and of the H–bound OH group (Das et al., 2002). The transmittance band at 2,919 cm^–1^ is due to the vibrational mode of C–H stretching. The peak clearly observed at 1,634 cm^–1^ was assigned to the bending vibration of water molecules (Li et al., 2005). The band observed at 2,352 cm^–1^ was assigned to the existence of CO_2_ molecule in air. The presence of reducing sugars and terpenoids in plant extract plays a role in reduction of metal ions and formation of metal NPs (Marchiol et al., 2014). It has been documented that the proteins present in the plant extract act as a capping agent (Niraimathi et al., 2013). The possible mechanism of capping of the metal NPs in green synthesis is because of the interactions of various phytocompounds present in sufficiently high concentration such as flavanones, terpenoids, alkaloids, and so on, with the particles (Ali et al., 2015). Therefore, the FTIR results indicate that the phytocompounds of root extract of *W. somnifera* were responsible for the synthesis, as well as stabilization and/or stabilization of TiO_2_ NPs.

Thermal decomposition performance was carried out in nitrogen gas from 25°C to 900°C and displayed in Figure 1D. The TGA curve readily showed three steps of weight loss, as confirmed by the DTG curve. The weight loss up to 900°C of the as-prepared sample is approximately 5.6%. The first weight loss up to 300°C was ascribed to desorption of physically adsorbed/retained water and volatility of the alcohol and acetone solvent. The second weight loss between 300°C and 650°C reflected the elimination of chemically bounded water and the thermal decomposition of plant organic residues. Above 650°C, the weight loss became fairly insignificant, indicating the formation of anatase TiO_2_ NPs (Yodyingyong et al., 2011).

**FIGURE 2:**
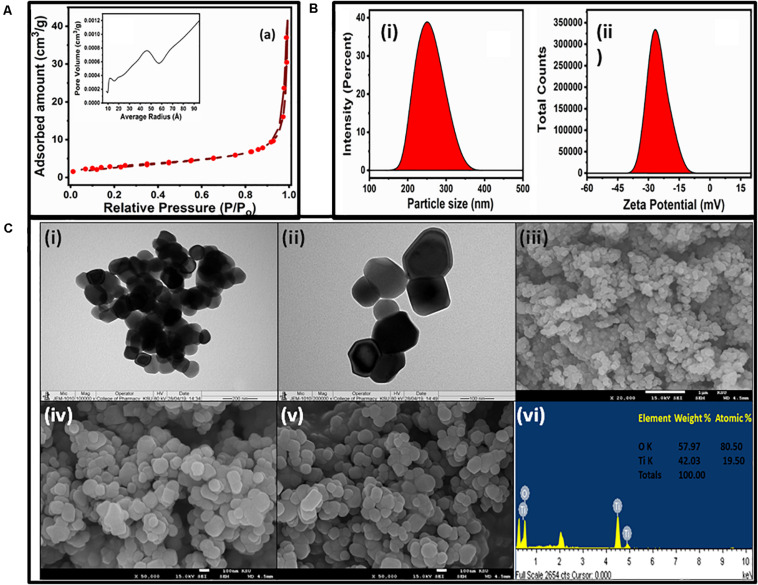
**(A)** N_2_ adsorption–desorption isotherms and pore-size distribution curves (inset) of the TiO_2_ NPs. **(B)** Hydrodynamic size **(i)** and zeta potential **(ii)** of the TiO_2_ NPs. **(C)** TEM **(i,ii)**, SEM **(iii–v)**, and EDS **(vi)** micrographs of TiO_2_ NPs.

The adsorption–desorption isotherm and pore width distribution are presented in Figure 2A. It shows that NPs have type IV isotherm with hysteresis loops of H3 (Yu et al., 2010). Type IV adsorption–desorption isotherms indicated the existence of mesoporous entities (4–20 nm, average pore diameter of approximately 24.53 nm), which provide broad surface for biological activity. The plot of differential volumes versus pore diameters indicated a narrower pore-size distribution. It exhibited a specific surface area of about 10.70 m^2^/g with specific pore volume of 0.065 cm^3^/g.

The DLS graph of TiO_2_ NPs are shown in Figure 2B. The dynamic light-scattering technique is an efficient method to measure particle diameter and zeta potential in the original grain size distribution. The DLS study indicated the TiO_2_ NPs to have an average size of 247 nm, with an intercept of 0.918 and a high–low polydispersity index of 0.631 (Figure 2Bi). The zeta potential of the TiO_2_ NPs was found to be −24 mV (Figure 2Bii) that also evidenced for the stability of TiO_2_ NPs. This negative potential was due to a good capping layer of the extract surrounding the NPs (Ravichandran et al., 2016). The size obtained from DLS analysis was greater than those observed in TEM and SEM because the hydrodynamic diameter was considered in the DLS measurement.

The size and morphology of TiO_2_ NPs were characterized by TEM and SEM-EDS. Typical TEM images are shown in Figures 2Ci,ii. The TEM results confirmed that the TiO_2_ NPs were aggregates of spherical and square NPs, and the size of TiO_2_ NPs ranged from 50 to 90 nm. The tendency for agglomeration was caused by van der Waals interactions between individual particles. The TEM image at lower magnification depicted a spherical structure while at high magnification shows the spherical and square shape. The SEM images and EDS spectrum of the sample were taken at 2,000 × magnification, which is shown in Figures 2Ciii–vi. It revealed that the overall surface morphology was spherical in shape, porous in nature, and variable in size. The size of NPs varied from 40 to 100 nm, which agreed with the TEM result. The elemental compositions of TiO_2_ NPs have been analyzed by EDS, as shown in Figure 2Cvi. The elemental compositions revealed that Ti and O were present nearly as per the expected stoichiometry (inset Figure 2C vi). The EDS spectrum showed the presence of Ti and O peaks around 4.5 and 0.5 KeV, respectively. The EDS spectrum analysis also revealed that the fabricated TiO_2_ NPs were free from any other impurities.

### Antibiofilm Activity of TiO_2_ NPs

**FIGURE 3:**
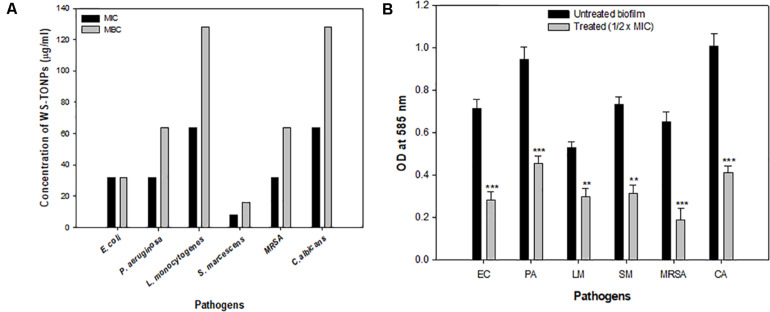
**(A)** Antibacterial activity of TiO_2_ NPs. Bar represents MIC and MBC values in μg/mL of TiO_2_ NPs against test pathogens. **(B)** Effect of 0.5 × MIC of TiO_2_ NPs on biofilm formation. **significance at *p* ≤ 0.01, and ***significance at *p* ≤ 0.005.

Minimum inhibitory concentration of synthesized TiO_2_ NPs was determined against all test pathogens. Among bacteria, the highest MIC value of 64 μg/mL was exhibited by *L. monocytogenes*, whereas *S. marcescens* with MIC of 8 μg/mL was found to be the most sensitive, as shown in Figure 3A. Titanium oxide NPs failed to show any bactericidal activity at concentrations lower than 32 μg/mL toward *E. coli*, *P. aeruginosa*, and MRSA. Hence, the 32 μg/mL concentration was considered as the MIC for these three bacteria. Titanium oxide NPs were inhibitory to *C. albicans* at a 64 μg/mL concentration. The MBC values of TiO_2_ NPs against test pathogens ranged from 16 to 128 μg/mL as depicted in Figure 3A. Similar antimicrobial potential of green synthesized TiO_2_ NPs was reported previously (Jayaseelan et al., 2013; Rajkumari et al., 2019).

Drug resistance poses an enormous threat to public health and environment. Biofilms immensely contribute to acquiring and disseminating resistance. These are well-organized multicellular aggregated communities enclosed in a self-secreted envelope of EPSs that prevents antimicrobial diffusion. Further, close proximity and high density of the cells facilitate the transfer of genetic material among the biofilm-making microbes, which is a hotspot for drug resistance (Balcázar et al., 2015). Biofilm-related infections account for spreading various diseases, especially in cases related to medical implants. As far as the food-based industry is concerned, biofilm formation on food matrixes, food contact surfaces, or machines can lead to persistent infections, leading to food-borne diseases (Galié et al., 2018). Hence, microbial biofilm control using eco-friendly NPs is a promising approach in preventing the spread of infection and diseases.

In the current investigation, 0.5 × MIC of TiO_2_ NPs was considered to explore its biofilm inhibitory potential against a range of bacteria and *C. albicans*. The results of the biofilm inhibition assay are summarized in Figure 3B. Among bacteria, the highest inhibition of 71% was recorded in MRSA, and the lowest was recorded in *L. monocytogenes* (43%). Inhibition of biofilm formation in *E. coli*, *P. aeruginosa*, and *S. marcescens* was observed to be 60%, 51%, and 57%, respectively, compared with the untreated control. Biofilm formation in *C. albicans* was significantly reduced with 32 μg/mL concentration of TiO_2_ NP treatment. The percent reduction in biofilm formation was recorded to be 59% over the untreated *Candida* biofilm, as shown in Figure 3B. This is probably the first report demonstrating TiO_2_ NPs’ broad-spectrum biofilm inhibitory activity. Previously, TiO_2_ NP have been reported to demonstrate significant biofilm reduction in oral bacteria *Streptococcus mitis* (Khan et al., 2016).

**FIGURE 4:**
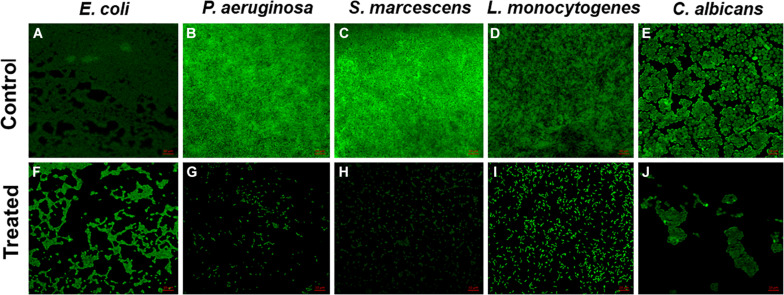
Confocal laser scanning microscopy images. Untreated biofilms of **(A)** *E. coli*, **(B)** *P. aeruginosa*, **(C)** *S. marcescens*, **(D)** *L. monocytogenes*, and **(E)** *C. albicans*; inhibition of biofilm by 0.5 × MIC **(F)** *E. coli*, **(G)** *P. aeruginosa*, **(H)** *S. marcescens*, **(I)** *L. monocytogenes*, and **(J)** *C. albicans*.

The *in vitro* microtiter plate biofilm inhibition assay results were further confirmed by microscopic analysis. CLSM images showed obliterated biofilm structures in all the pathogens treated with their respective sub-MICs of TiO_2_ NPs (Figure 4). The untreated control strains showed dense clusters of microbial aggregation, and cells exhibited normal morphology. In contrast, altered biofilm structures were observed in NP-treated cells. Microbial cells were scattered and less dense compared with the untreated control.

**FIGURE 5:**
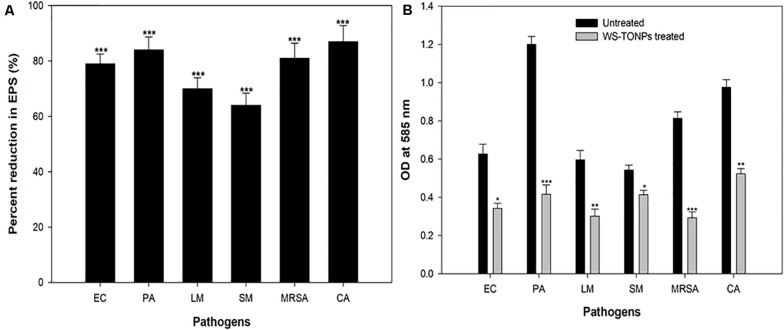
**(A)** Inhibitory effect of TiO_2_ NPs on EPS production by test pathogens. ***significance at *p* ≤ 0.005. **(B)** Biofilm disruption effect of TiO_2_ NPs. *Significance at *p* ≤ 0.05, **significance at *p* ≤ 0.01, and ***significance at *p* ≤ 0.005.

Exopolysaccharides are a very important component of the biofilm architecture, as they not only provide structure stability but also protect cells from environmental stresses, entry of antimicrobials, and disinfection (Flemming et al., 2007). Therefore, intrusion in EPS production will certainly have adverse effects on the biofilm-forming capability of the pathogens. We found statistically significant reduction in EPS production of the test pathogens in the presence of 0.5 × MICs of synthesized NPs, as shown in Figure 5A. Among the Gram-positive bacteria, MRSA and *L. monocytogenes* showed 81% and 70% decrease in EPS production, respectively, whereas the group of Gram-negative bacteria, namely, *E. coli*, *P. aeruginosa*, and *S. marcescens*, exhibited 79%, 84%, and 64% reduction, respectively. Our findings agree with the researchers who reported reduced EPS production by *P. aeruginosa* upon treatment with 31.25 μg/mL concentration of TiO_2_ NPs synthesized from the leaves of *A. barbadensis* (Rajkumari et al., 2019).

### Inhibition of Mature Preformed Biofilms

Mature biofilms are hard to eradicate using chemical agents, owing to the drug resistance that biofilm imparts to the microbial cells residing in this mode. The efficacy of TiO_2_ NPs in eradicating mature preformed biofilms of the bacterial and fungal pathogens was examined. Figure 5B shows histograms depicting the TiO_2_ NP–induced reduction in the preformed biofilms of the test pathogens. Our data reveal statistically significant disruption of 45%, 65%, 49%, 64%, 24%, and 46% in *E. coli*, *P. aeruginosa*, *L. monocytogenes*, MRSA, *S. marcescens*, and *C. albicans* preformed biofilms, respectively. Biofilm matrix comprising different kinds of biomolecules, such as peptides, polysaccharides, and nucleic acids, is responsible for forming the barrier against antimicrobial agents (Costerton et al., 1999). The assay findings clearly demonstrate that the synthesized NPs could breach the barrier and disrupt the biofilm. For the first time, we reported the broad-spectrum obliteration of bacterial and *Candida* mature biofilms by TiO_2_ NPs.

### Mechanism of Biofilm Inhibition

#### Protein Leakage Assay

**FIGURE 6:**
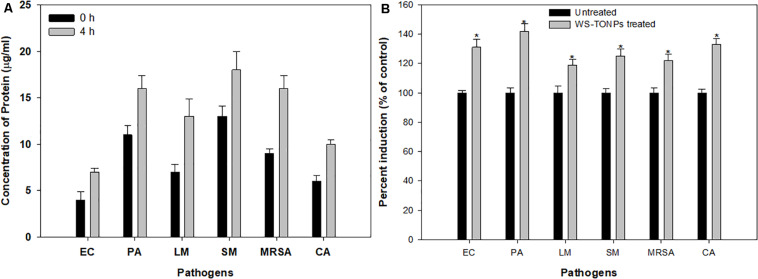
**(A)** Protein leakage from test pathogens treated with 0.5 × MIC of TiO_2_ NPs. **(B)** Induction of ROS generation in test pathogens treated with 0.5 × MIC of TiO_2_ NPs. *Significance at *p* < 0.05.

We performed the leakage assay to study TiO_2_ NPs’ possible mode of action on inhibiting biofilm formation. Significant upsurge in the released protein content in NP-treated samples was observed after 4 h of incubation (Figure 6A). These increased protein content suggested that NPs lysed and destructed the cell wall of the test pathogens, leading to cell death and eventually inhibiting biofilm formation. In a recent report, similar protein leakage due to changes in the membrane permeability of *E. coli* and *S. aureus* cells treated with TiO_2_ NPs has been demonstrated (Khater et al., 2020).

#### ROS Generation Studies

The relative amount of ROS generated in the presence and absence of TiO_2_ NPs is summarized in Figure 6B. The considerable effect on intercellular ROS production was recorded upon exposure to 0.5 × MIC of TiO_2_ NPs. The highest ROS increase of 42% was recorded for *P. aeruginosa*, followed by *C. albicans* (33%), *E. coli* (31%), *S. marcescens* (25%), and MRSA (22%), and the least was recorded for *L. monocytogenes* (19%). ROS generation is one of the chief mechanisms by which NPs interfere with normal microbial cell functions. The ROS-scavenging enzymes present in the bacterial cell neutralize ROS generated in untreated cells, whereas in the NP-treated bacterial cells, enhanced ROS levels overpower the ROS-scavenging enzymes, leading to oxidative stress, which results in lipid peroxidation and, eventually, cell death (Kulshrestha et al., 2017). Hence, we expected that the intracellular ROS produced by TiO_2_ NP–treated cells of the test pathogens overpowered the cellular antioxidant defense system and caused cell mortality by inducing oxidative stress. Our results also showed that ROS generation was lower in Gram-positive bacteria, possibly due to their thick cell wall (Al-Shabib et al., 2018a).

### Cytotoxicity Studies

#### MTT and NRU Assay

**FIGURE 7:**
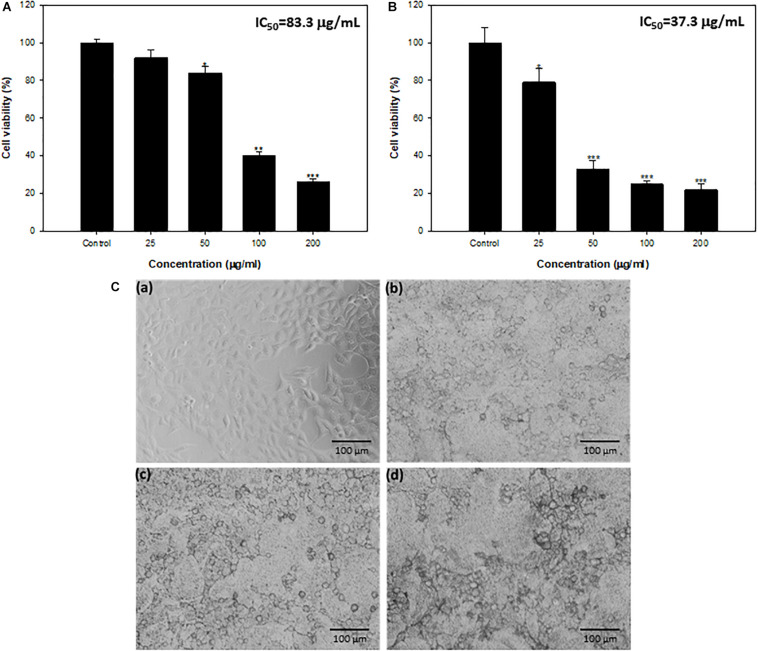
**(A)** Cytotoxicity determination by MTT assay against HepG2 cell lines. **(B)** Cytotoxicity determination by NRU assay against HepG2 cell lines. *Significance at *p* ≤ 0.05, **significance at *p* ≤ 0.01, and ***significance at *p* ≤ 0.005. **(C)** HepG2 cells exposed to TiO_2_ NPs for 24 h. **(a)** Control, **(b)** 50 μg/mL, **(c)** 100 μg/mL, and **(d)** 200 μg/mL.

Anticancer properties of synthesized TiO_2_ NPs against HepG2 were assayed by MTT and NRU. Effect on HepG2 cells exposed to different TiO_2_ NP concentrations (25–200 μg/mL) is depicted in terms of cell viability (%) in Figures 7A,B. Titanium oxide NPs induced concentration-dependent decrease in cell viability of HepG2. Cell viability was recorded as 92%, 88%, 40%, and 26% at 25, 50, 100, and 200 μg/mL concentrations, respectively, using MTT assay (Figure 7A). Further, TiO_2_ NPs demonstrated insignificant toxicity against non-tumorigenic HEK293 (human embryonic kidney) cells; more than 93% cells were viable at the highest tested concentration of 200 μg/mL (Supplementary Figure S1). Similar concentration-dependent cell viability reduction was recorded with NRU assay. The viability of HepG2 cells was found to be 79%, 33%, 25%, and 22% at 25, 50, 100, and 200 μg/mL TiO_2_ NP concentrations, in comparison with untreated cells (100%) (Figure 7B). The IC_50_ values obtained were 83.3 and 37.3 μg/mL, respectively, through the MTT and NRU assays.

Microscopic analysis also showed concentration-dependent changes in the morphology of HepG2 cells. Most of the cells at 50–200 μg/mL concentration lost normal morphology, appeared round in shape, decreased cell density, and highly reduced cell adhesion capacity (Figure 7C).

Both these assays are sensitive and integrated to measure the anticancer activity of synthesized NPs. The MTT assay evaluates mitochondrial function, whereas NRU assesses lysosomal functions (Ahamed et al., 2017). Possible mechanism of cytotoxicity exhibited by TiO_2_ NPs could be the enhanced production of ROS as reported by several workers. Increased ROS levels get the better of the antioxidant defense system, leading to oxidative stress, which triggers apoptosis causing cell shrinkage (Jin et al., 2008; Sha et al., 2011). Similar concentration-dependent HepG2 cell cytotoxicity by TiO_2_ NPs synthesized from *Bacillus cereus* has also been demonstrated (Sunkar et al., 2014). Bacterial biofilms formed in human intestines have been reported to sustain and trigger colorectal cancer progression. Molecular processes involved in the interaction of carcinogenic factors formed by pathogens, their biofilms, and the host’s response in colorectal cancer initiation and progression have also emerged (Mirzaei et al., 2020). Furthermore, the aggregation of bacteria in biofilms was reported to cause injuries and inflammation of intestinal epithelial tissues, thus aggravating the cancer (Gao et al., 2015). Experimental evidence has suggested that initiation and development of cancer are a consequence of pro-oncogenic properties of biofilms formed by invasive pathogenic bacteria (Johnson et al., 2015). Because microbial biofilm is reported to play a critical etiologic role in cancer development, biofilm inhibition, along with TiO_2_ NP–induced cytotoxicity, is an important finding.

## Conclusion

In summary, we achieved successful phytomediated synthesis of green TiO_2_ NPs from root extract of *W. somnifera*, assessed the broad-spectrum biofilm inhibitory activity against bacterial and fungal pathogens, and evaluated HepG2 cytotoxicity. Root extracts of *W. somnifera* acted as a reducing, capping, and stabilizing agent for the synthesis of TiO_2_ NPs. Synthesized TiO_2_ NPs demonstrated significant biofilm inhibition and destruction of preformed biofilms of *P. aeruginosa*, *C. albicans*, *E. coli*, *S. marcescens*, MRSA, and *L. monocytogenes*. Impaired biofilm formation could plausibly be due to the cell death caused by intracellular ROS generation in TiO_2_ NP–treated pathogens. Furthermore, different concentrations of TiO_2_ NPs induced significant reduction in HepG2 cancer cell viability. Thus, the synthesized green NPs could prove as effective agents in the treatment of biofilm-based bacterial and fungal infections. Further, these NPs could also have a positive impact in the food industry by reducing environmental biofouling. Moreover, because biofilm formation sustains and triggers cancer development, the cytotoxicity of these NPs against human hepatic cancer cell line HepG2, along with its broad-spectrum biofilm inhibition, can be exploited to prevent and control cancers, with respect to pharmacologic treatments. Finally, more molecular and animal model investigations are requisite to uncover the exact mechanisms.

## Data Availability Statement

The raw data supporting the conclusions of this article will be made available by the authors, without undue reservation.

## Author Contributions

NA-S, FH, FQ, and NA conceived and designed the experiments. FH, FQ, AK, AA, MA, SN, PA, NA, and JK performed the experiments. FH, FQ, NA, MA, SN, PA, TA, JK, AK, and SS performed the experiments and analyzed the data. NA-S, FH, FQ, JK, PA, SN, TA, and AA wrote the manuscript. All authors reviewed and approved the manuscript.

## Conflict of Interest

The authors declare that the research was conducted in the absence of any commercial or financial relationships that could be construed as a potential conflict of interest.
