# Supplementary material for: Phyto-Mediated Synthesis of Porous Titanium Dioxide Nanoparticles From Withania somnifera Root Extract: Broad-Spectrum Attenuation of Biofilm and Cytotoxic Properties Against HepG2 Cell Lines
Source: Front Microbiol. 2020 Jul 28;11:1680. doi: 10.3389/fmicb.2020.01680 (PMC7399045; doi:10.3389/fmicb.2020.01680)
Supplement: Supplementary file 1 [file Data_Sheet_1.docx]

**Supplementary material**

Figure S1. Cytotoxicity assessment by MTT assay in HEK293 cell line
